# CoCl_2_, a Mimic of Hypoxia, Induces Formation of Polyploid Giant Cells with Stem Characteristics in Colon Cancer

**DOI:** 10.1371/journal.pone.0099143

**Published:** 2014-06-16

**Authors:** Laura M. Lopez-Sánchez, Carla Jimenez, Araceli Valverde, Vanessa Hernandez, Jon Peñarando, Antonio Martinez, Chary Lopez-Pedrera, Juan R. Muñoz-Castañeda, Juan R. De la Haba-Rodríguez, Enrique Aranda, Antonio Rodriguez-Ariza

**Affiliations:** 1 Oncology Department, Maimonides Institute of Biomedical Research (IMIBIC), Reina Sofía Hospital, University of Córdoba, Córdoba, Spain; 2 Spanish Cancer Network (RTICC), Instituto de Salud Carlos III, Madrid, Spain; 3 Research Unit, Maimonides Institute of Biomedical Research (IMIBIC), Reina Sofía Hospital, University of Córdoba, Córdoba, Spain; Rush University Medical Center, United States of America

## Abstract

The induction of polyploidy is considered the reproductive end of cells, but there is evidence that polyploid giant cancer cells (PGCCs) contribute to cell repopulation during tumor relapse. However, the role of these cells in the development, progression and response to therapy in colon cancer remains undefined. Therefore, the main objective of this study was to investigate the generation of PGCCs in colon cancer cells and identify mechanisms of formation. Treatment of HCT-116 and Caco-2 colon cancer cells with the hypoxia mimic CoCl_2_ induced the formation of cells with larger cell and nuclear size (PGCCs), while the cells with normal morphology were selectively eliminated. Cytometric analysis showed that CoCl_2_ treatment induced G2 cell cycle arrest and the generation of a polyploid cell subpopulation with increased cellular DNA content. Polyploidy of hypoxia-induced PGCCs was confirmed by FISH analysis. Furthermore, CoCl_2_ treatment effectively induced the stabilization of HIF-1α, the differential expression of a truncated form of p53 (p47) and decreased levels of cyclin D1, indicating molecular mechanisms associated with cell cycle arrest at G2. Generation of PGCCs also contributed to expansion of a cell subpopulation with cancer stem cells (CSCs) characteristics, as indicated by colonosphere formation assays, and enhanced chemoresistance to 5-fluorouracil and oxaliplatin. In conclusion, the pharmacological induction of hypoxia in colon cancer cells causes the formation of PGCCs, the expansion of a cell subpopulation with CSC characteristics and chemoresistance. The molecular mechanisms involved, including the stabilization of HIF-1 α, the involvement of p53/p47 isoform and cell cycle arrest at G2, suggest novel targets to prevent tumor relapse and treatment failure in colon cancer.

## Introduction

Colorectal cancer (CRC) is the second most common cancer with 1,234,000 cases worldwide in 2008 according to GLOBOCAN [1]. CRC accounts for 13% of all cancers and almost 1000 new CRC cases were diagnosed in 2012 in Europe [2], where is the third most frequent cancer and after lung cancer it is the second most frequent cause of death [3]. Although death rates from CRC have decreased slightly from 1990 to the present, and despite advances in detection and surgical treatment, there is no known cure for metastatic CRC, and the 5-year survival rate of these patients is disappointingly low (about 8%). The existence of a relatively rare slowly proliferating or resting cell subpopulation, highly resistant to drugs, with similar properties to stem cells and known as cancer stem cells (CSCs), has been proposed as one main cause of the alarming inefficiency of standard cancer therapies [4], [5]. During the last decade, it has been shown that these CSCs are able to proliferate and produce the whole tumor mass. This has led to propose a model of CSCs, or tumor hierarchical model, in which tumor cells are heterogeneous, and only a small cell population, which is at the top of the hierarchical pyramid, is responsible for initiating and maintaining tumor growth [5]. However, more recent studies suggest that cancer stemness can be acquired by changing gene expression programs and therefore CSC biology must shift from a rigid hierarchical to a more flexible model [6], [7]. CSCs are much more resistant than differentiated tumor cells to the therapies that are used in clinic [4], [8] and, although the treatment is able to effectively remove most of the tumor cells and tumor volume decreases, CSCs are not affected and once the therapy stops they are able to resume tumor growth and differentiation, explaining events as tumor recurrence. Thus, it has been shown that the risk of recurrence of CRC is proportional to the expression in the primary tumor of genes specific for intestinal stem cells which also identify a CSC population in the tumor [9]. Besides, CSCs seem to play an important role in the dissemination process, tumor dormancy and metastasis [10].

Hypoxia is one of the most important pathological features of the solid tumors, because it is the result of an imbalance between proliferation of tumor cells and the oxygen supply [11]. Tumor hypoxia not only represents a major problem affecting therapeutic efforts, but there is experimental evidence that constitutes a physiological selective pressure promoting tumor aggressiveness [12]. Importantly, hypoxia is associated with the maintenance and formation of CSCs [11], [13], promoting their phenotype and tumorigenesis [14]. Many of the cellular responses to hypoxia are mediated through changes in gene expression regulated by hypoxia inducible factor (HIF-1α), which has become a very attractive target for developing new cancer therapies [11]. Under normoxic conditions HIF-1α protein is continuously degraded after hydroxylation by prolyl hydroxylases of two key proline residues in its oxygen dependent degradation domain [15]. However, under hypoxic conditions HIF-1α is stabilized, translocates to the nucleus and, upon binding to HIF-1β subunit, forms an active transcription factor capable of activating the expression of target genes facilitating cellular adaptation to hypoxia [13]. Cobalt chloride (CoCl_2_) is a mimetic agent used *in vitro* to induce cellular responses mediated by hypoxia. It is believed that CoCl_2_ stabilizes HIF-1α by inhibiting prolyl hydroxylase enzymes [16].

It has been suggested that, similarly to normal stem cells, the local microenvironment is critical for maintaining the survival of CSCs in their “niche” where reception of the specific molecular signals regulate their proliferation and differentiation [17]. Surprisingly, although several studies have recently demonstrated an important role of endothelial cells (ECs) and perivascular niche in the regulation of normal stem cells and CSCs, other studies indicate that hypoxia also plays a key role [17]. The importance of a perivascular niche in regulating CSCs on one hand and the role of hypoxia in the other may seem at first sight paradoxical. Nevertheless, ECs in the tumor microenvironment can undergo functional alterations that lead to the generation of a hypoxic microenvironment. Thus, glioblastoma CSCs are found in intimate contact with the aberrant tumour vasculature [18]. Recently it was reported that hypoxia can select polyploid giant cancer cells, (PGCCs) that contribute actively to tumor growth by generating CSCs [19]. In fact, the majority of human tumors are cellular heterogeneous [20], and for over a century, pathologists have observed giant cell tumors, significantly more often after some form of therapeutic intervention [19]. These cells contribute to tumor heterogeneity but most of their functions are not yet defined. Although the induction of polyploidy has been traditionally considered the reproductive end of cells, there is evidence that giant cells contribute to cell repopulation during tumor recurrence [21], [22].

Although there are data on the generation of PGCCs in ovarian and breast cancer, the role of these cells in the development, progression and response to therapy in CRC still remains undefined. Therefore, the main objective of this study was to investigate the *in vitro* generation PGCCs in colon cancer cells and identify mechanisms of formation.

## Materials and Methods

### Cell Culture and colonosphere formation assay

Caco-2 cells (ECACC, Salisbury, UK) were grown in MEM with Earle's salts (PAA Laboratories GmbH, Pasching, Austria) containing 15% fetal bovine serum. HCT-116 cells (DSMZ, Braunschweig, Germany) were grown in McCoy's 5A medium (Biowest, Nuaillé, France) containing 10% fetal bovine serum (PAA Laboratories). Culture media were supplemented with 2 mM glutamine, 1% non-essential amino acids, penicillin (100 U/ml), streptomycin (100 µg/ml) and amphotericin B (2.5 µg/ml). Cells were maintained in a humidified atmosphere at 37°C and 5% CO_2_. A fresh stock solution 0.4 M cobalt chloride (CoCl_2_) was prepared in water and added to the medium to obtain desired final concentrations.

For the colonosphere formation assay, after treatment with different doses of CoCl_2_ for 48 h, cells were tripsinized, counted and re-seeded at clonal density (1 cell/µl) in 96-well plate with ultra-low attachment surface (Costar, Corning, NY, USA) with serum free Dulbecco's MEM Nutrient Mixture F+12 Ham medium supplemented with 10 ng/ml basic fibroblast growth factor, 20 ng/ml epidermal growth factor and 1% v/v methylcellulose (R&D Systems, Minneapolis, MN, USA) to prevent cell aggregation. The supplements were freshly added every 2–3 days and the number and size of formed colonospheres were evaluated by optical microscopy on day 7 after seeding. Secondary colonospheres were formed from the cell population obtained after trypsin-EDTA disaggregation of primary spheres and seeded at clonal density and cultured as described above. To obtain a sufficient cell number for secondary colonosphere formation, primary colonospheres were seeded for 7 days in 6-well ultra-low attachment plates.

### Western Blotting

After 6 and 48 h of treatment, grown cells were harvested with cold PBS and centrifuged at 300× g, 4°C for 5 minutes. The cell pellet was incubated for 15 minutes on ice with 1 ml lysis buffer (50 mM Tris-HCl (pH 7.4), 150 mM NaCl, 5 mM ethylenediamine tetraacetic acid (EDTA), 1 mM ethyleneglycol tetraacetic acid (EGTA), 1.5 mM MgCl_2_, 10% glycerol, 1% NP40, 0.1 M dithiothreitol (DTT), 0.1 M phenylmethylsulfonyl fluoride (PMSF), 1% v/v protease inhibitor cocktail (SERVA, Heidelberg, Germany) and 1% v/v phosphatase inhibitor cocktails 2 and 3 (Sigma-Aldrich) and centrifuged at 15,000× g for 15 minutes at 4°C. Total protein concentration was quantified by a standard Bradford assay using the colorimetric reagent from BioRad Laboratories (Hercules, CA, USA). Proteins (12.5 µg) were separated onto SDS polyacrylamide gels using a 4–12% Bis-Tris gradient gels in the BioRad Criterion System and transferred to a nitrocellulose membrane (BioRad Laboratories, Hercules, CA, USA). Then, the membranes were blocked for 1 h at room temperature with 5% non-fat milk in Tris-buffered saline with 0.2% Tween-20 followed by incubation with secondary antibody conjugated with horseradish peroxidase, and detection by chemiluminescent reaction with the ECL Plus Western Blotting Detection System or ECL Advance Western Blotting Detection Kit (GE Healthcare Life Sciences, Little Chal- font, UK). Images were captured on a ChemiDoc XRS Imaging System (BioRad Hercules, CA, USA) and densitometric analyses of protein bands detected were performed with image-J software (NIH).

Antibodies used were as follows: monoclonal anti-HIF-1a, polyclonal anti-p53 and polyclonal anti-actin and secondary antibodies conjugated with horseradish peroxidase were from Santa Cruz Biotechnology (Santa Cruz, CA, USA). Monoclonal anti-cyclin D1 was from Cell Signaling (Beverly, MA, USA).

### Cell proliferation

Cells were seeded in 96-well plates (4,000 cells/well) and treated with CoCl_2_ as described above. After 48 h of treatment, cells were then exposed for 72 h to different doses of 5-fluorouracil or oxaliplatin and cell proliferation was assayed using the XTT Cell Proliferation Assay Kit (Roche, Basel. Switzerland) following the manufacturer's instructions. During the assay, XTT tetrazolium salt is reduced to an orange colored formazan dye by dehydrogenases and reductases enzymes in metabolically active cells, which was detected espectrophotometrically (450 to 655 nm) using an ImarkTM Microplate Reader (Biorad, Hercules, CA, USA). In each assay, cell proliferation was expressed as the percentage of untreated cells.

### Cell cycle analysis

Cells (0.5–1×10^6^ cells) were trypsinized and resuspended in PBS. Ice-cold 100% ethanol was added in a drop-wise manner while gently vortexing and incubated for 20 minutes at room temperature. Samples were centrifuged at 100× g for 5 minutes, resuspended in PBS containing 50 µg/ml propidium iodide plus 100 µg/ml RNase A and incubated for 20 minutes at room temperature protected from light. Analysis and measurement of propidium iodide fluorescence were performed on a FACSCalibur (BD Biosciences) flow cytometer.

### Immunofluorescence analysis

For DAPI staining, cells were permeabilizated and fixated by incubation with 50% methanol for 5 minutes at room temperature followed by 100% methanol for 20 minutes at −20°C. Then, cells were incubated again with 50% methanol for 5 minutes at room temperature and finally kept in PBS. At this point the cells were incubated with 2 µM 4′,6-diamidino-2-phenylindole dihydrochloride (DAPI) in PBS for 5 minutes at room temperature for fluorescent staining of DNA content. Cells were maintained in PBS and observed under a fluorescence microscope (Nikon, Tokyo, Japan). Obtained images were digitized and nuclear area was quantified using image-J software (National Institutes of Health).

The analysis of polyploidy by Fluorescent In Situ Hybridization (FISH) was performed in the laboratory of Molecular Genetics from Hospital Reina Sofia using the AneuVysion kit (Abbott Molecular, Inc./Vysis, Downers Grove, IL) following standard recommended protocol. Cells were centrifugated at 100× g for 10 minutes and incubated in trypsin-EDTA for 20 minutes in a 37°C water bath. Then, cell pellet was again centrifuged for 5 minutes, and incubated in 0,56% KCl for 20 minutes at 37°C. After addition of few drops of Carnoy's solution (methanol∶glacial acetic acid [3∶1]), samples were centrifuged, resuspended and kept in Carnoy's solution at 4°C for at least 30 minutes or until ready to perform FISH. The supernatant was discarded and the cells were diluted in 200 µl of Carnoy's solution. The cells were smeared in two areas on the same slide, one area for hybridization with probes LSI (21 and 13), and the other area for hybridization with probes CEP (X/Y and 18). The slides were covered by a cover glass and introduced in a HYBrite system (Abbott) overnight, with a melting temperature of 74°C for 5 minutes followed by hybridization at 27°C for 20 h. Next day, the slides were washed with a wash solution (0.4× SSC/03% NP-40) at 70°C for 2 min followed by 2× SSC/0.1% NP-40 at room temperature for 1 minute. Finally, slides were air dried, counterstained with DAPI, and analyzed under a fluorescence microscope (Nikon, Tokyo, Japan).

### Statistical analysis

Results are expressed as means as mean ± SD and are representative of three separate experiments. Statistical comparisons were performed using a two-tailed Student's t test. Differences were considered significant at p<0.05.

## Results

### CoCl_2_ treatment induces the formation of PGCCs in colon cancer cells

**Figure 1:**
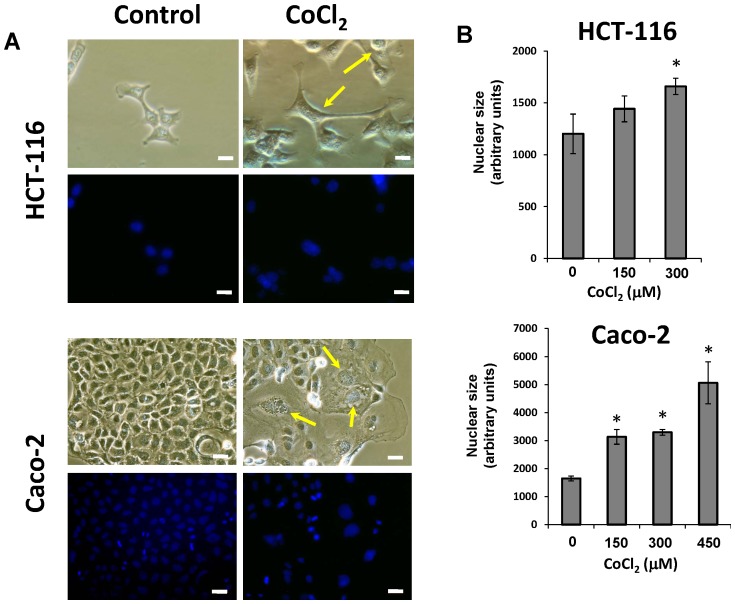
Morphological changes of colon cancer cells after treatment with CoCl_2_. A) Both HCT-116 and Caco-2 cell lines were treated with 300 µM CoCl_2_ for 48 hours. All photomicrographs were obtained using the same final magnification (×200). Scale bar corresponds to 20 microns. Staining of cell nuclei with DAPI was performed to evaluate the average nuclear size (Final magnification, ×200). Scale bars correspond to 20 microns. B) Changes in nuclear size after CoCl_2_ treatment. Data represent the mean ± SD of three independent experiments (*p<0.05, compared with the control).

When HCT-116 and Caco-2 colon cancer cell lines were cultured under normal conditions some large cells with enlarged nuclei (PGCCs) were sporadically observed. However, when the cells were treated with CoCl_2_ these PGCCs clearly increased in number, while cells with normal morphology were selectively eliminated (Figure 1A). The PGCCs induced by CoCl_2_ treatment were 3–10 times larger than normal cells, and with a distinctive morphology depending on cell line (Figure 1A). Thus in HCT-116 cells the treatment with CoCl_2_ generated PGCCs with cytoplasmic extensions reminiscent of cells of neuronal type (Figure 1A), whereas in Caco-2 cells the induced PGCCs were rounded and had no branches (Fig. 1A). The increase in nuclear size after treatment also varied between the two cell lines, and although a clear increase was observed in both cell lines, the nuclear enlargement was more pronounced in the case of Caco-2 cells (Figure 1B).

### PGCCs formation in colon cancer cells is associated with cell cycle alterations and polyploidy

**Figure 2:**
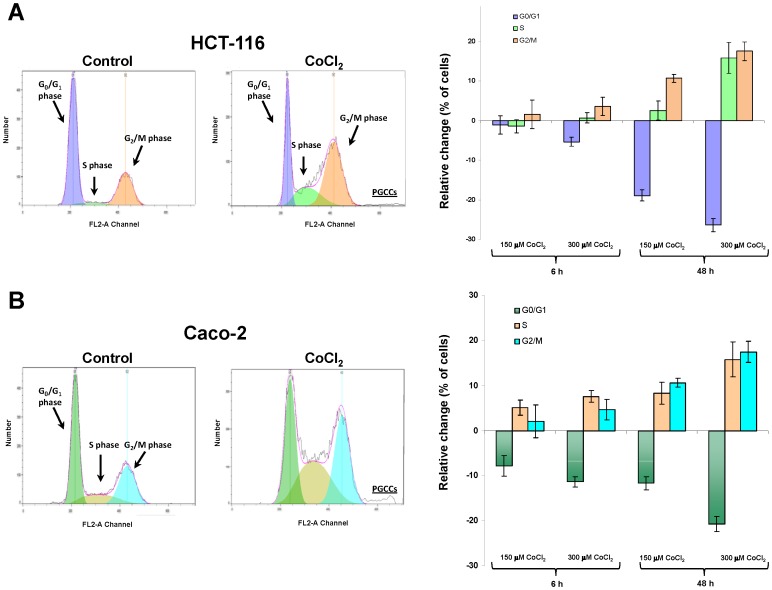
Alteration of cell cycle in colon cancer exposed to CoCl_2_. Both HCT-116 (A) and Caco-2 (B) cells were treated with the indicated doses of CoCl_2_ for 6 or 48 hours. Next, a cell cycle analysis was performed by flow cytometry. The bar graphs on the right show the relative changes to control cells in the percentage of cells in different phases of the cell cycle. Data represent the mean ± SD of three independent cultures.

**Figure 3:**
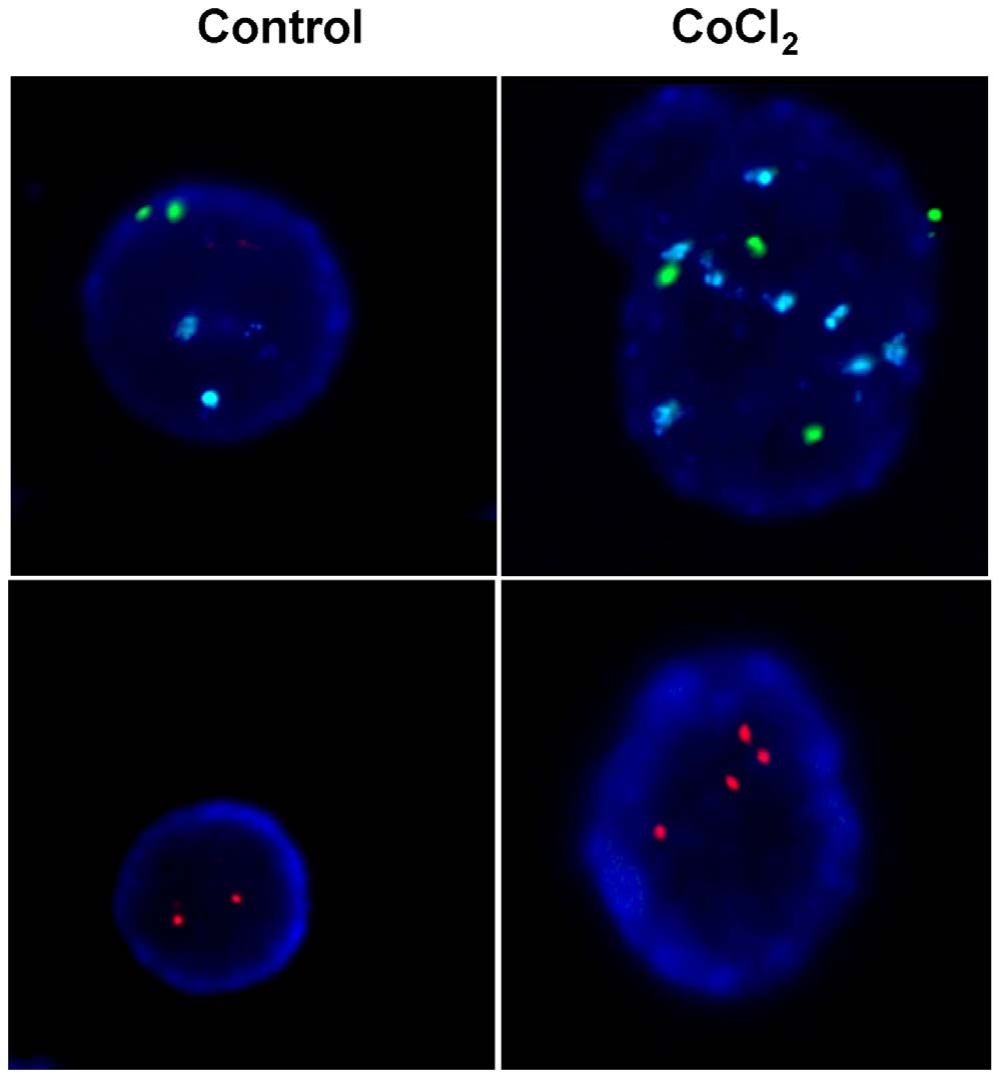
Confirmation of induction of cell polyploidy in colon cancer cells after treatment with CoCl_2_. HCT-116 cells were cultured in the presence of 300 µM CoCl_2_ and then a FISH analysis using probes for chromosomes X (green), 18 (blue) and 21 (red) was performed.

To further explore the mechanisms responsible for the induction of PGCCs, cell cycle was analyzed using flow cytometry. In both cell lines CoCl_2_ treatment caused clear alterations in various phases of the cell cycle (Figure 2). A clear reduction in the number of cells in G1 phase, along with an increase of cells in S and G2 phases was observed. Furthermore, flow cytometric analysis confirmed that the treatment with CoCl_2_ that mimics hypoxia is capable of generating a cell subpopulation with a high increase in DNA content and therefore corresponding to PGCCs. The polyploid nature of these cells was confirmed by fluorescence in situ hybridization (FISH) to detect the presence of multiple copies of DNA in individual PGCCs. For Caco-2 cells FISH analyses yielded inconclusive results, possibly because this cell line already has a severely altered karyotype. However, as can be seen in Figure 3, while HCT-116 control cells showed normal nuclear size and markers indicated normal disomy for chromosomes X (green), 18 (blue) and 21 (red), treatment with CoCl_2_, induced PGCCs showing an increased nuclear size accompanied by polyploidy for the three chromosomal markers used.

### Molecular mechanisms involved in the formation of PGCCs in colon cancer

**Figure 4:**
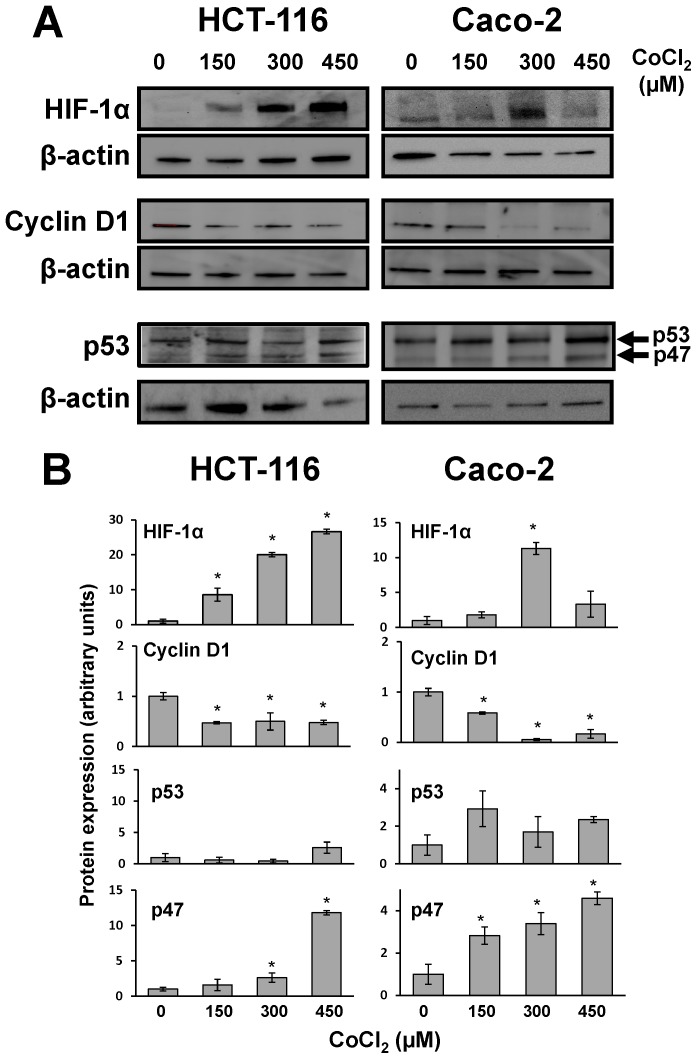
HIF-1α, p53, and cyclin D1 expression in colon cancer cells treated with CoCl_2_. A) HCT-116 and Caco-2 cells were treated with the indicated doses of CoCl_2_ for 6 hours and then the expression of HIF-1α, p53 and cyclin D1 proteins was evaluated using specific antibodies. B) The corresponding densitometric analyses of the protein bands detected in the immunoblots and normalized to the signal of β-actin are also shown. Data are means ± SD of three independent experiments. (*p<0.05, compared with the control).

A series of experiments were next performed to analyze the possible molecular mechanisms involved in the generation of PGCCs in colon cancer cells. First, the expression of HIF-1α protein was analyzed to confirm that the treatment with CoCl_2_ can effectively induce its stabilization. As shown in Figure 4, although the expression of HIF-1α was undetectable in control cells, the treatment with CoCl_2_ induced in both cell lines, and in a dose-dependent manner, an increase in the expression levels of this protein. Therefore, the mimic of hypoxia in colon cancer cells is associated with alterations in cell cycle and the generation of PGCCs.

On the other hand, the expression of cyclin D1 is essential for the cell cycle to progress from G1 to S phase, a point where the cell is already committed to completing a new round of cell division. Once the cell “decides” to begin this S phase, high levels of cyclin D1 are downregulated to allow DNA synthesis. If conditions continue to allow cell growth, cyclin D1 levels increase again during the G2 phase. However, if the cell cycle is compromised at this point, cyclin D1 levels remain low. As shown in Figure 4, lower levels of cyclin D1 observed upon treatment of HCT-116 or Caco-2 cells with CoCl_2_ also suggested a cell cycle arrest at G2, confirming results obtained in the flow cytometry analysis.

**Figure 5:**
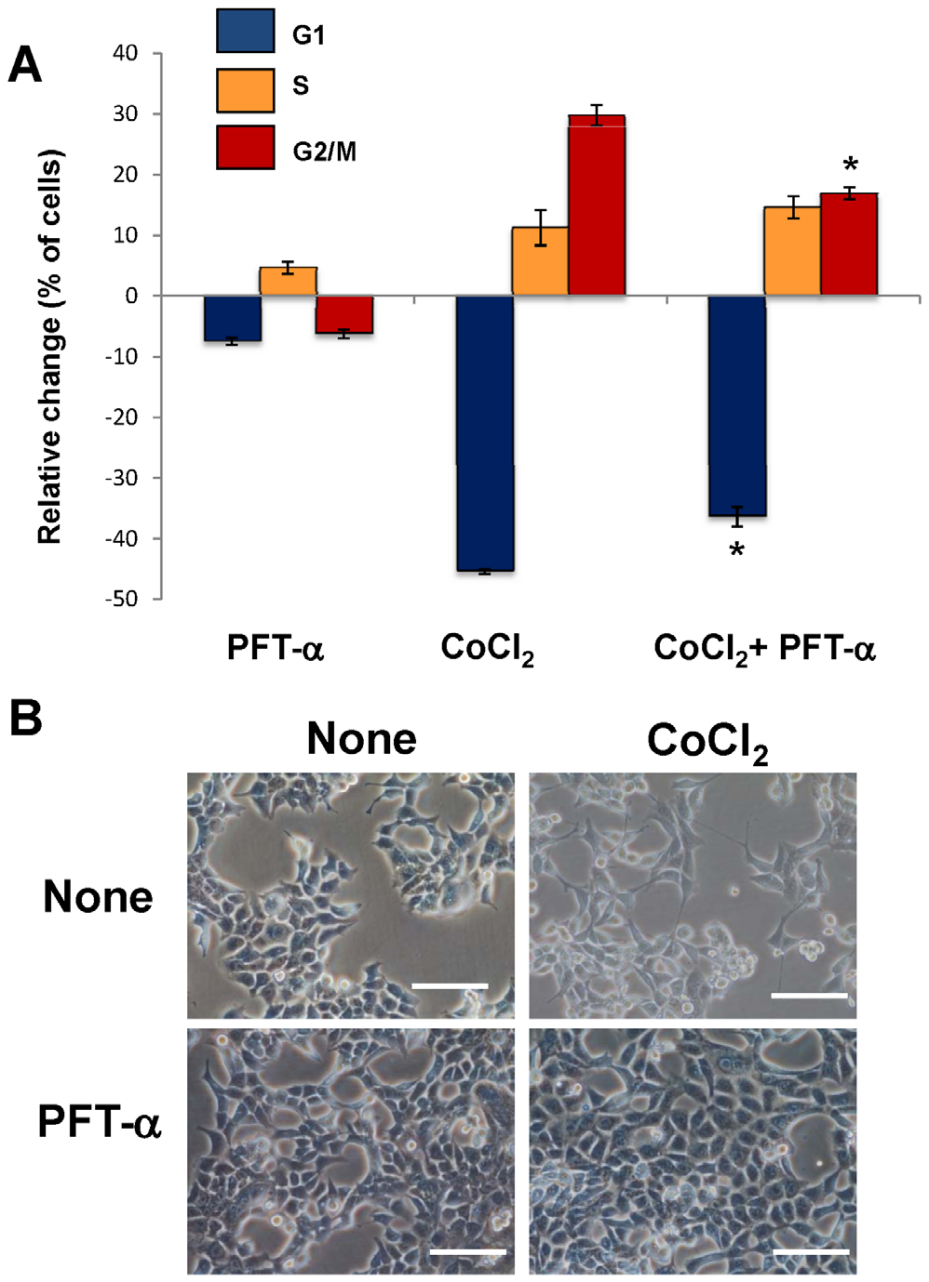
Effect of p53 inhibition on hypoxia-induced cell cycle alterations and the formation of PGCCs in colon cancer cells. HCT-116 cells were treated with 300 µM CoCl_2_ in the presence or in the absence of 10 µM pifithrin-α (PFT-α), which is a specific inhibitor of the transcriptional activity of p53. Next, a cell cycle analysis was performed by flow cytometry.(A) Data represent the mean ± SD of three independent cultures. (*p<0.05, compared with cells exposed to CoCl_2_ in the absence of PFT-α), and (B) changes in cell morphology were examined using visible microscopy. Scale bars correspond to 50 microns.

It is known that p53 plays an important role in regulating cell cycle progression at G1 and G2 checkpoints. Specifically, in response to DNA damage, activation of molecular pathways regulated by p53, leads to cell cycle arrest at G1 [23]. Furthermore, more recently it has been reported that expression of a p53 isoform lacking the first 39 amino acids and termed p47, is involved in cell cycle arrest at G2 in response to different cell stress conditions, including endoplasmic reticulum stress, unfolded protein response and hypoxia [24]. Therefore, we next analyzed the expression of both p53 isoforms in colon cancer cells after treatment with CoCl_2_. As shown in Figure 4, treatment with CoCl_2_ did not alter the expression of full length protein p53 (p53FL) but induced the expression of the truncated isoform (p53/p47). These results suggest that mimic of hypoxia in colon cancer cells induces the expression of p53/p47 isoform negatively regulating the cell cycle progression at G2. Therefore, the role of p53/p47 was further explored by treating colon cancer cells with pifithrin-α (PFT-α), which specifically suppress p53-mediated transactivation. Treatment of Caco-2 cells with PFT-α caused an unexpected cytotoxicity which precluded subsequent morphological and cell cycle analyses. However, and as shown in Figure 5A, treatment of HCT-116 cells with 10 µM PFT-α reduced the impact of CoCl_2_ on cell cycle Furthermore, this pretreatment, which specifically blocks the transcriptional activity of p53, abrogated the formation of PGCCs induced by CoCl_2_ in HCT-116 cells (Figure 5B). Therefore, transcriptionally active p53/p47 is necessary for the hypoxia-induced cell cycle alterations and the formation of PGCCs in colon cancer cells.

### PGCCs generation in colon cancer is associated with an increase in the subpopulation of cancer stem cells

It has been suggested that hypoxia generated PGCCs may actively contribute to the tumor growth by generation of CSCs [19]. Therefore we decided to analyze then formation of colonospheres *in vitro*, which is a functional assay of the self-renewal capbility of CSCs. To this end, after enrichment in PGCCs by treatment with CoCl_2_ for 48 h, surviving cells were cultured at clonal density with serum free medium in low-adherence plates that prevent cellular adhesion. Under these conditions, differentiated epithelial tumor cells die by anoikis and only a subpopulation of tumor undifferentiated cells with stem characteristics (CSCs) survives, which is capable of generating tumorospheres (colonospheres) in suspension by self-renewal. Previous experiments using lipophilic fluorescent stains were performed to confirm that individual colonospheres were derived from single cells. Thus, mixing of equal numbers of DiI (Red)- or DiO (Green)-labelled cells prior to performing the colonosphere formation assay resulted in the formation of spheres containing only one or the other label (Figure S1).

**Figure 6:**
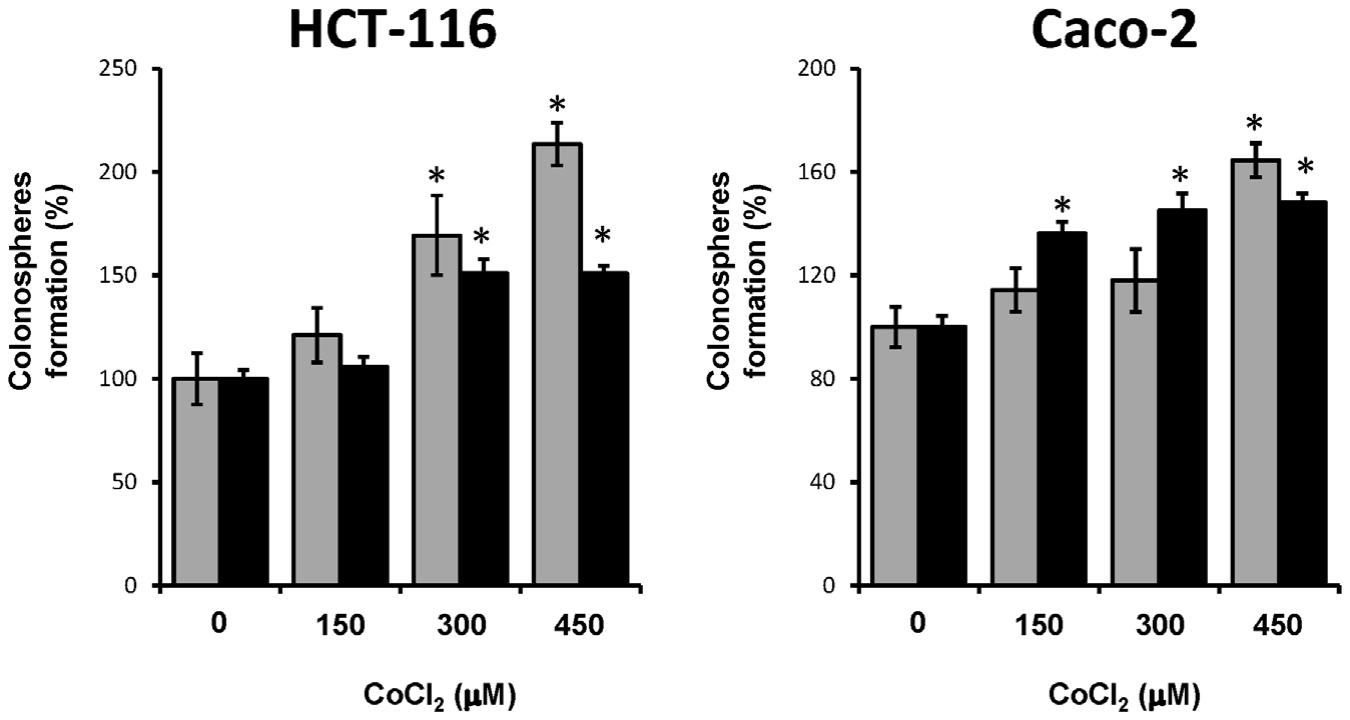
PGCCs enrichment in colon cancer cells increases colonosphere formation capability. After enrichment in PGCCs by treatment for 48 µM CoCl_2_, surviving cells were cultured at clonal density with serum free medium in low-adherence plates and the number of primary formed colonospheres was evaluated after seven days. Primary colonospheres were disaggregated and the resulting cell population was cultured again at clonal density for the formation of secondary colonospheres. Graphics show the numbers of primary (grey bars) and secondary (black bars) colonospheres formed by HCT-116 and Caco-2 cells after each treatment. The data represent the mean ± SD of three independent cultures (*p<0,05, compared to control).

**Figure 7:**
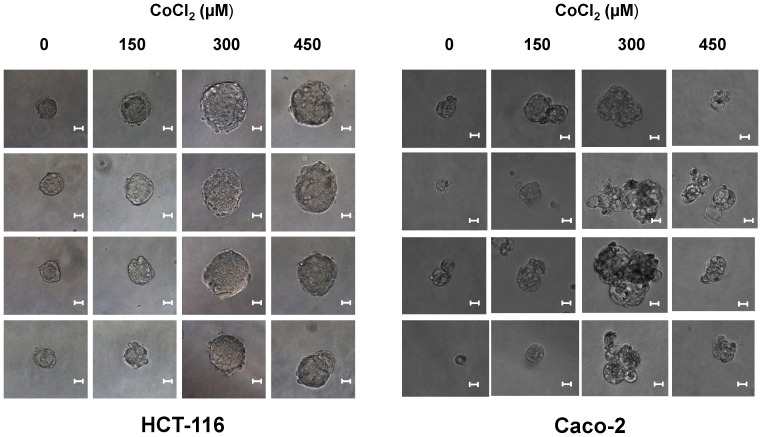
Increase in size of secondary colonospheres formed by colon cancer cells enriched in PGCCs. The secondary colonosphere formation assay was performed as described in Figure 6 legend and under Materials and Methods. (Final magnification, ×200). Scale bar corresponds to 10 microns.

As shown in Figure 6, pretreatment with CoCl_2_ and subsequent enrichment in PGGCs increased in both HCT-116 and Caco-2 cells the capability to form colonospheres, suggesting that the generation of PGCCs contributes to the expansion of the CSC subpopulation in both cell lines. Next, the formed colonospheres were disaggregated and the resulting cell population was cultured again at clonal density for the formation of secondary colonospheres. (Figure 6). Cells derived from primary colonospheres formed by CoCl_2_-treated colon cancer cells retained a higher ability to form colonospheres, confirming a superior self-renewal capacity. Furthermore, secondary colonospheres derived from colonospheres formed by cells subjected to hypoxia were significantly larger in size compared with those derived from control cells (Figure 7). This increase in size corroborated that CSCs generated in a cell population enriched in PGCCs also possessed a greater ability for self-renewal, and therefore they had more marked stemness characteristics [25].

### The generation of PGCCs in colon cancer cells is associated with resistance to 5-fluorouracil and oxaliplatin

**Figure 8:**
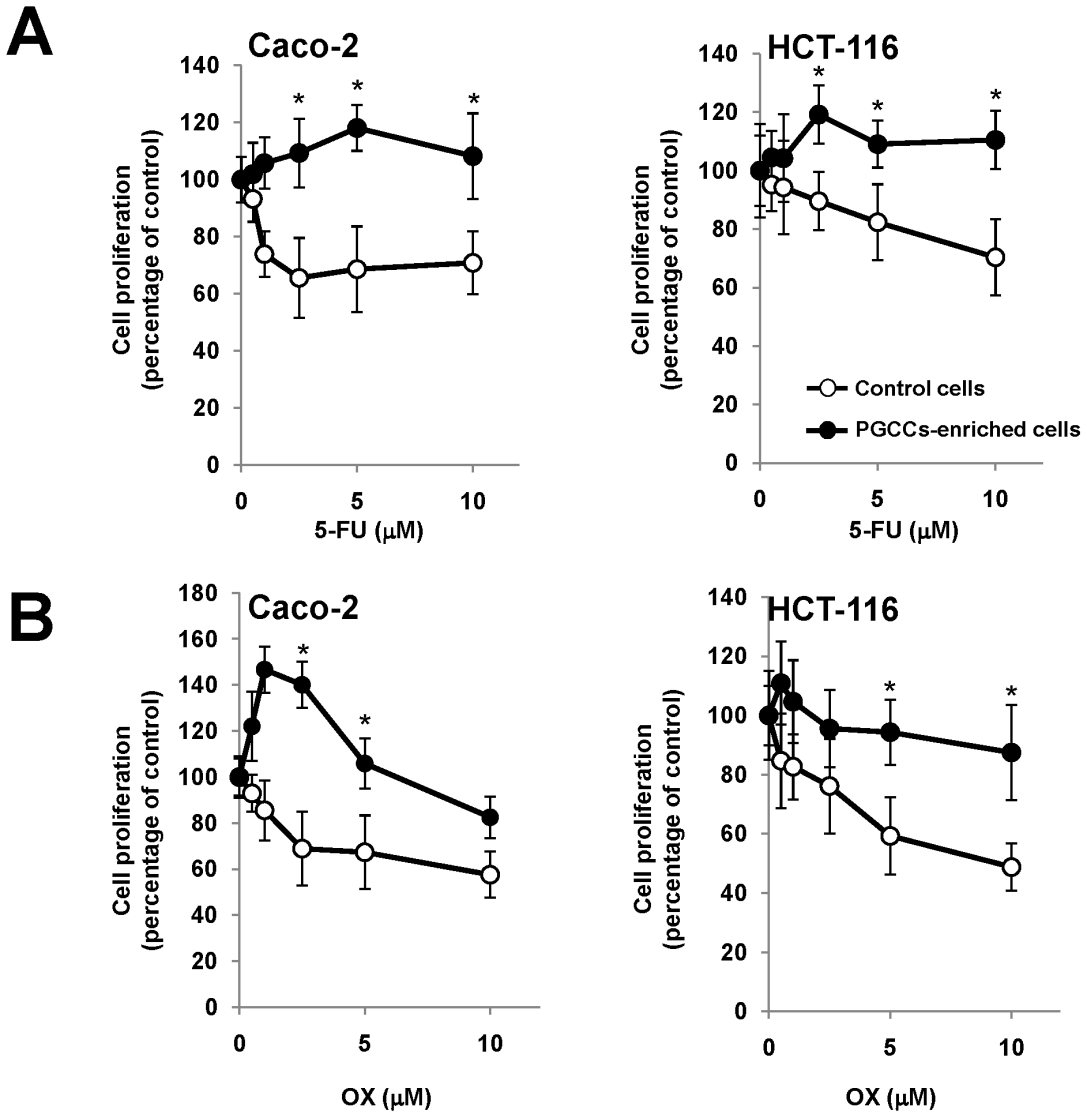
Colon cancer cells enriched in PGCCs show chemoresistance. Both HCT-116 and Caco-2 cells were preincubated for 48 hours in the presence or absence of CoCl_2_ for PGCC enrichment and then they were exposed for 72 h to different doses of 5-fluorouracil (A) or oxaliplatin (B). Cell proliferation is shown as percentage of control cells unexposed to chemotherapeutic drugs. The data represent the mean ± SD of three independent experiments (* p<0.05, compared to cells without CoCl_2_ treatment).

Compared to more normoxic tumors, tumors with a higher hypoxic fraction are more resistant to radiotherapy and chemotherapy [12]. Also, both PGCCs [19] as CSCs [5] have been associated with resistance to chemotherapy. Therefore we next decided to analyze in colon cancer cell cultures enriched in PGCCs the antiproliferative activity of 5-fluorouracil and oxaliplatin, two commonly used drugs in the chemotherapy of colorectal cancer. As shown in Figure 8, those cell cultures that had been enriched in PGCCs by treatment with CoCl_2_, showed an increased resistance to the antiproliferative effect of both drugs.

## Discussion

Hypoxia in tumors has long been associated with increased tumor aggressiveness, worse prognosis and greater resistance to radiotherapy and chemotherapy [26]. In this study we demonstrated that mimicking hypoxia in vitro by using CoCl_2_, which stabilizes HIF-1α protein by inhibiting its degradation, is capable of generating PGCCs in colon cancer cell culture. PGCCs induced by treatment with CoCl_2_ are stable and possessed distinctive morphology but their generation under physiological *in vivo* hypoxic conditions remains to be determined. In our experiments, the morphology of PGCCs induced by hypoxia varied between HCT-116 and Caco-2 cells (Figure 1). This dependence on cell line coincides with the study of Zhang et al [19] where PGCCs derived tumor cell lines HEY (ovarian) and MDA -MB-231 (breast) had a neuronal-like morphology, whereas PGGCs derived from SKOV3 cells (ovary), showed a rounded morphology without cytoplasmic extensions or branches. In our study, the morphology of the PGCCs derived from HCT-116 cells coincides with those from HEY and MDA -MB-231 cells, while those generated in Caco- 2 were more similar to those described for SKOV3 cells. It should be emphasized that HEY, MDA -MB- 231 and HCT-116 are markedly invasive cell lines, being representative of the epithelial mesenchymal transition (EMT) process which suffer some subpopulations of tumor cells [27]. Therefore, or results suggest that the genetic program that controls EMT in tumor cells also might be an important factor in the generation of PGCCs with higher capacity of infiltration and spreading.

Our results also indicated that the formation of PGCCs in colon cancer cells is associated with changes in cell cycle and subsequent polyploidy (Figures 2 and 3). Unicellular prokaryotic and eukaryotic cells divide by amitotic processes. In complex eukaryotic cells, although mitosis prevails, well-documented changes in the mitotic cell cycle occurs to achieve cell growth and development under stressful circumstances. Among these variations is the endoreplication process, a variation of normal mitotic cell cycle which involves multiple rounds of DNA replication without the participation of the step of mitosis [28]. Tumor giant cells observed by pathologists have traditionally been considered as inert from the point of view of tumor repopulation. However, new data indicate that these cells are capable of generating clonogenic progeny by asymmetric division and budding [19]. Thus, a process has been described in which cells escape mitotic catastrophe-induced cell death by becoming PGCCs that, before they die, give rise to several cells via nuclear budding and asymmetric cytokinesis. This mode of cell division in cancer has been termed neosis, and has been related to the origin of cancer stem cells [29], [30]. Furthermore, it has been shown in some tumors that these PGCCs retain reproductive potential participating in the development of chemo- and radio-resistance [21], [31]. In this regard, DNA endoreduplication and reversible polyploidy generating clonogenic escape cells has been identified as a mechanism which can account for tumor relapse after initial efficient chemotherapy [32].

Our cytometric analyses indicate that the generation of PGCCs in colon cancer cells is associated with a specific cell cycle arrest at G2. Additionally, protein expression analysis (Figure 4) confirmed that the stabilization of HIF-1α was accompanied by lower levels of cyclin D1, which also indicates a cell cycle arrest in the transition from the S to G2 phase of cell cycle [33]. The p53 protein is a transcription factor that plays a pivotal role in cell cycle regulation, being considered a tumor suppressor as its frequent inactivation, or its signaling alteration in tumor cells, enables the evasion from the strict cell growth control that occurs in normal cells. Many types of stress and cellular damage lead to p53 activation, and one of the most perplexing questions is how cells can differentiate p53 activation in order to respond to the cell cycle arrest in G1 or G2, senescence, repair or apoptosis. However, it is reported that both ER stress, the unfolded protein response (UPR) and hypoxic conditions are associated with an arrest at G2 cell cycle regulated by the p47 isoform expression of p53 [24]. Our results suggest that, under hypoxic conditions that enrich colon cancer cells in PGCCs, the cell cycle arrest at G2 is also mediated by the expression of this p53 isoform. Tumor hypoxia is an opportunity for the development of new targeted therapies in cancer, and in this respect our results support that the relationship between hypoxia, ER and UPR may constitute novel potential therapeutic targets for the treatment of colon cancer.

Several studies have provided evidence that ECs and the perivascular niche play an important role in the generation and maintenance of CSCs [17]. Interestingly other recent studies has related hypoxia to the niche of CSCs. Thus, the in vivo HIF-1α deletion decreases the ability of tumor spread mediated by CSCs in leukemia [34]. Also, in brain and pancreatic tumors hypoxia promotes the expansion of CSCs through the activation of HIF-1α [35], [36]. It has also been shown that hypoxia, by means of HIF-1α activation, is capable of maintaining CSCs phenotype in colon cancer cells [37]. The notion that hypoxia is involved in the generation and maintenance of CSCs is also supported by the results obtained in our study. The induction of hypoxic response in the two cell lines used and the generation of PGCCs increased the capacity to form colonospheres (Figure 6), a functional assay for the ability of self-renewal that is characteristic of CSCs [25]. Furthermore, the fact that those cultures treated with CoCl_2_ were also able to generate secondary colonospheres of larger size (Figure 7) indicates that hypoxia and the generation PGCCs are directly related to the expansion and survival of a cell population with increased capacity for self-renewal and thus more tumorigenic.

It has been suggested that the perivascular niche within the tumor microenvironment can undergo functional alterations that lead to the generation of a hypoxic microenvironment [17]. Tumor neovasculature often develops rapidly, resulting in structural and functional abnormalities ultimately leading to reduced oxygen transport. Also, it has been reported that the generation of intratumoral hypoxia after antiangiogenic treatment increase the population of CSC in breast cancer, thus providing a potential explanation for the limited clinical effectiveness of these anticancer drugs [38]. It is still unclear whether there is a perivascular niche for CSCs in colon cancer, although it has been recently shown that ECs are capable of promoting the phenotype of CSCs in intestinal tumors [39]. Emerging evidence has implicated a number of vascular-derived factors that can regulate CSCs [18] and it is tempting to speculate that the generation of nitric oxide (NO) by endothelial nitric oxide synthase may be involved in this aspect. In fact, it has been described the ability of NO to stabilize HIF-1α [40], and NO donors such as DETA-NONOate and L-nitrosocysteine are capable of increasing the levels of HIF-α in HCT-116 cells (results not shown).

CSCs have also been associated with increased resistance to cancer therapy, so that, although the treatment is able to effectively eradicate most of the tumor cells and the tumor volume decreases, the CSCs are not affected and once the therapy ceases are able to resume growth and tumor differentiation, explaining events such as tumor recurrence [4]. Furthermore, the expression of HIF-1α has been also associated with resistance to 5-fluorouracil [41] and oxaliplatin [42], two commonly used drugs in standard care of colorectal cancer. Hypoxia and the generation of PGCCs may therefore play an important role in the chemoresistance that eventually develops most of colorectal cancer patients. Our results support this hypothesis, since both 5-fluorouracil and oxaliplatin showed reduced antiproliferative activity in colon cancer cells cultures that had been pretreated with CoCl_2_ and enriched in PGCCs (Figure 8).

The generation of PGCCs in a hypoxic and possibly perivascular niche in colon tumors may constitute a reservoir of CSCs which eventually may repopulate a tumor after showing response to therapy. These mechanisms could also participate in the phenomena of resistance and/or recurrence observed in antiangiogenic therapy in colon cancer. The molecular mechanisms involved in the hypoxic generation of PGCCs, including HIF-1α stabilization, involvement of p53/p47 isoform and cell cycle arrest in G2, point to novel therapeutic targets for the prevention of tumor recurrence and treatment failure in colon cancer.

## Supporting Information

Figure S1 **Formed colonospheres are derived from single cells.** Lipophilic fluorescent labeling were performed to confirm that individual colonospheres were derived from single cells. Equal numbers of DiI (Red)- or DiO (Green)-labelled cells were mixed prior to seeding at clonal density to perform the colonosphere formation assay, as described under Materials and Methods. The assay resulted in the formation of DiI (Red)- or DiO (Green)-labelled spheres, whereas mixed labeled colonospheres were not observed, thus confirming that tumorospheres are derived from single cells.
(TIF)
