# Supplementary figures and images for: CoCl2, a Mimic of Hypoxia, Induces Formation of Polyploid Giant Cells with Stem Characteristics in Colon Cancer
Source: PLoS One. 2014 Jun 16;9(6):e99143. doi: 10.1371/journal.pone.0099143 (PMC4059626; doi:10.1371/journal.pone.0099143)

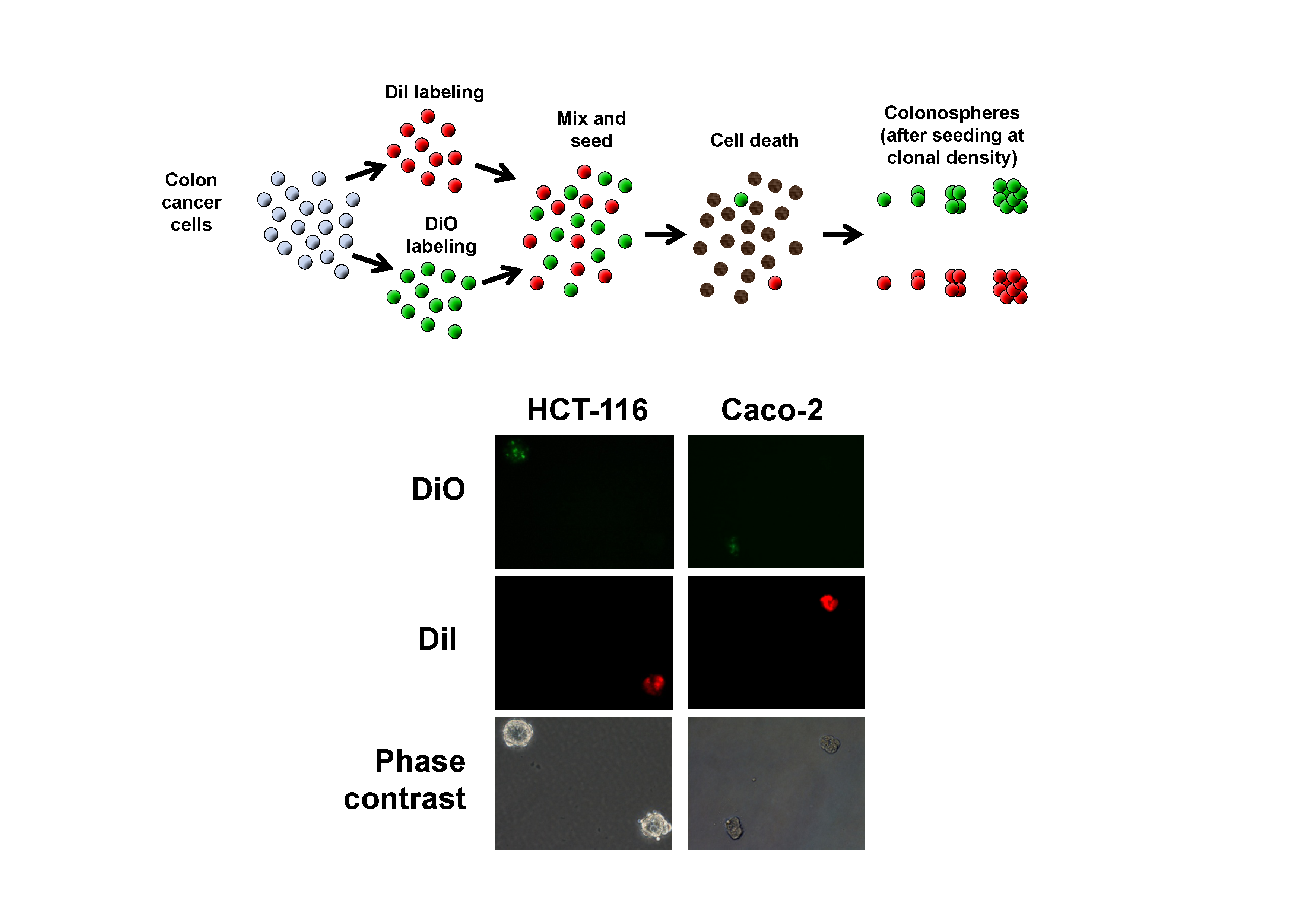

Supplement: Figure S1 — Formed colonospheres are derived from single cells. Lipophilic fluorescent labeling were performed to confirm that individual colonospheres were derived from single cells. Equal numbers of DiI (Red)- or DiO (Green)-labelled cells were mixed prior to seeding at clonal density to perform the colonosphere formation assay, as described under Materials and Methods. The assay resulted in the formation of DiI (Red)- or DiO (Green)-labelled spheres, whereas mixed labeled colonospheres were not observed, thus confirming that tumorospheres are derived from single cells. (TIF) [file pone.0099143.s001.tif]
